# Allegheny County Medical Society

**Published:** 1889-01

**Authors:** 


### ALLEGHENY COUNTY MEDICAL SOCIETY.

*Special Meeting, December 18th, 1888.*

J. Chris Lange, M.D., Vice-President, in the Chair.

Dr. Duff reported a case of

### PAINFUL JOINTS, WITH MIXED PAPILLARY AND PUSTULAR ERUPTION.

I was called this evening to see a girl thirteen years of age. Two years ago she was down with inflammatory rheumatism. She came home last Wednesday from school, complaining of slight pain in one of her ankles. There was no perceptible swelling, her mother stated, but the pain increased slightly until Saturday, when the other ankle became affected. On Sunday a papillary eruption appeared on the first ankle, and on yesterday morning a very free eruption appeared on the other ankle. Yesterday afternoon the wrists and elbow on the right side began to swell and pain her, and simultaneously with the swelling, this papillary eruption appeared on both the joint of the elbow and of the wrist, and about this time the eruption became pustular upon the ankles. This afternoon she was taken worse, and simultaneously with the appearance of the pain, the swelling on the other arm and papillary eruption appeared. This evening I found her with a temperature of

104°, pulse 120, pustular eruption on both ankles, and upon the elbow and forearm on the left side there is a mixture of papillary and pustular eruption; whilst on the right arm it is papillary. No local application had been made to the parts except a little camphor liniment.

Dr. Davis reported a case of

AMPUTATION THROUGH DOUBTFUL TISSUES.

About two weeks since a strong, able-bodied Pole was admitted to Mercy Hospital. He was admitted on Tuesday. On the preceding Friday, while coupling cars in a coal works near Punxsutawney, his arm was caught in the coupling and slightly crushed. He brought a letter from the doctor who attended him at the time, Dr. Williams, of Brooksville, or Punxsutawney, stating that the blood supply to the hand had no doubt been entirely cut off, and that he had urged amputation, but that the man had positively refused to have this done, and preferred to go to the city and get advice. The bandage on his arm, I presume, had been none too tight; when I saw it the hand was perfectly black. On looking at the arm, every evidence of gangrene was present. The arm was swollen to almost twice its normal size, with the peculiarly marked discoloration of progressive mortification, with the blistering down near the elbow. The line of demarcation had extended over the top of the shoulder. The resident at the hospital, who had seen him two hours before, assured me that when he had noticed it, it was not within at least four or five inches of the shoulder, and yet in two hours it had advanced to the shoulder with a high elevation, so that by passing the finger over from the healthy tissue to the diseased, you could readily discern the line of demarcation. The man's temperature was about 105°, his countenance anxious, his whole appearance that of one who had suffered an extreme shock to the system, and in whom disease was progressing rapidly. His condition was really critical, so critical that I felt that all hope of his life was surely gone. I called in some of the other gentlemen of the Hospital, and we concluded, at all events, not to let the man die with that black arm, as an amputation was really the most we had to hope for. I amputated the arm at the middle by the circular method, cutting through tissue absolutely black, cutting down through the fatty tissue of the arm and down to the muscular parts, the muscles having become not as yet thoroughly involved. I removed the arm, and then applied almost boiling hot water, and then bichloride solution, 1 to 1,000. I pressed out with the bandage as firmly as I could from the shoulder down all this material, and then filled the conical cavity full of iodoform, put a piece of cotton around, and left it. The man is rapidly improving, and will recover. I know that usually the surgeon who would have done this with any expectation of the recovery of the patient would have been considered very ignorant indeed; but I believe recovery was owing to the powerful antiseptics used, and to the use of the boiling water and the solution of bichloride.

Dr. Murdock: A very interesting case, I think, has been reported by Dr. Davis, and one that is instructive to us all. The old rule in surgery in regard to amputations in such cases was that in gangrene which arose from a constitutional cause, such as is the case in senile gangrene or as is the case where gangrene attacks a patient suffering from diabetes, to wait until the line of demarcation was formed. So also in some cases of gangrene resulting from local causes, such as frost-bite, the rule was to wait until the line of demarcation was formed; but in cases of injury, like the one related by Dr. Davis, I think that the rule was never thoroughly observed by the best surgeons, and that even the older surgeons advocated amputation in certain cases in vigorous patients while the gangrene was still progressing in cases of injury. But previous to the introduction of the antiseptic method of treatment, no surgeon would have thought of amputating through or so near dead tissue as Dr. Davis did in this case, and if he had done so without such treatment, he would not have been successful; the gangrene would have extended. For that reason this is a very interesting and instructive case.

There is another point which is very instructive and useful. Dr. Davis tells us that the arm was bandaged very tightly above the wound, but he does not think the bandage had anything to do with the gangrene. Whether it had or not, I wish to call attention to something that should be known and well observed by surgeons. It has been my fortune, since I have been connected with the Western Pennsylvania Hospital and before, to see patients brought to the hospital with tourniquets on limbs pressing entirely too tightly above the wound for the purpose of arresting hæmorrhage. I have seen several cases brought, where gangrene has resulted from the tightness of the bandage above the wound, applied in one case by a doctor, and in several other cases by those not professionals. I know of one case where a man was brought not far from here, with a not very severe wound of the leg. A tourniquet had been on the thigh twenty-four hours, and the limb was in a state of gangrene. This was owing entirely to the tightness of the bandage. I have known several such instances, and also instances where patients have been brought with very slight wounds, there being only wounds of veins, the tight bandage distending the veins. Now these frequent accidents justify the belief that if there was none of this tight bandaging, there would be more lives saved than lost as a result. I think more people are injured by the tourniquet applied to wounds than are benefited. This is my experience in these cases. This practice arises from faulty ideas in the minds of the people, which have emanated from the profession. The profession is accountable for the education which the people have, and the reason these cases are frequent is owing to the fact that the public cannot be instructed how to apply a bandage in case of a wound. It requires a great deal of experience to enable one to distinguish between the blood from an artery and from a vein.

The attempts to instruct men who are not professionals, who are not accustomed to observing severe injuries, result in more harm than good. The non-professional man, when he sees a wound, says to himself: "Now, this may be a wound of an artery or wound of a vein; I don't know exactly, but I will be on the safe side; I will put on the bandage above." Thus it happens that in every wound, severe or small, of vein or artery, the bandage is put on above the wound, and usually as tightly as it can be drawn. In nine cases out of ten these instructions result in injury. At a watering-place not far from this city, a little boy fell against a mirror and cut the veins of his wrist horizontally across. There were few gentlemen at the house, and the ladies were frightened; but there were some very intelligent gentlemen present. A tight bandage was put on above the elbow. The wound continued to bleed. Then they held the arm high up. It still continued to bleed. The boy bled for two hours, until the arrival of a physician. The physician stopped the hæmorrhage. He did it by removing the bandage. From like repeated experiences, I am of the opinion that the instruction being given to non-professionals results in more harm than help. If it be wise to attempt any teaching in the control of hæmorrhage, I would advise these rules: If the hæmorrhage be copious, and a bleeding point is seen in any wound, let it be covered by the finger point, and let this pressure continue until the arrival of a physician; if the bleeding be not copious, put a bandage, not above, nor below, but *upon* the wound.

DR. BATTEN: Dr. Davis was fortunate in the ending of his case. I had some experience in hospital gangrene in '62 and '63. Many of the wounded at that time were afflicted with gangrene. If the leg was wounded, the gangrene would extend around the leg and expose the vessels before amputation was resorted to; but the gangrene was not arrested by the operation. It extended. And in many cases re-amputation above the knee became necessary. I believe the knowledge that is being sown among the laity regarding the care of the wounded is more harmful than beneficial. Persons not accustomed to handling wounds are timid; if they do anything, it is as likely to be wrong as right.

Dr. Buchanan: I think Dr. Davis is giving rather more credit to the antiseptics than is justified by the case. No one, I think, can be a firmer believer in antiseptic treatment than myself, but I believe that Dr. Davis rather overrated the influence of his antiseptic agents in this case. Either the flaps in this amputation were dead or they were alive. The outcome of the case shows the flaps were alive; but the anti-septic agents are not to be credited as preserving them. Either the lymph channels were swarming with bacteria or they were not. If they had been so swarming, I do not think any application of antiseptic agents would have destroyed the micro-organisms they contained. I believe the man was suffering from the presence of the decomposing member, and that when Dr. Davis removed that, he removed the cause of the disease, and that probably if he had not used any antiseptic agent the irritation would have subsided as quickly.

I do not wish to be understood as saying that the outcome of the case would have been as favorable; in all probability, he would have had suppuration and trouble, but from the description of the case, I believe that the majority of men so affected would recover without antiseptic agents—not so nicely, indeed, but they would recover. I don't believe in giving more credit to the antiseptics than they deserve.

Dr. Duff: It is of importance, very frequently, that the non-professional who may witness an accident involving dangerous hæmorrhage shall possess the knowledge and skill to arrest this until the arrival of the surgeon. I have twice hastened to such cases to find the patients dead of hæmorrhage. It is to be regretted that the instructions now being given to engineers, firemen and the police are lacking in practicability; still they are, perhaps, better than no instructions,

Dr. Davis: In reply to Dr. Buchanan, I believe that until very recently, there is no authority for cutting through gangrenous parts. I do not know whether there were any bugs in the lymphatic system of this patient's arm or not. I am not much on bugs. I believe it was the hot water and the bichloride. The tissues were full of gas and the cutting pressed the bubbles of gas out, but whether there were any bacteria in them or not, I don't know.

Dr. J. J. Buchanan read a paper on an

UNUSUAL RESULT OF LONG-STANDING TARSAL CARIES.

The patient, a girl of sixteen years, came under my care in June last, with the following history: Family record free from tubercular or specific disease, health perfect till close of third year, when a bleb appeared over the outer aspect of the *os calcis*, which subsequently broke down and formed the extremity of a sinus leading to the bone. This sinus remained open for years, occasionally discharging detritus of carious bone, till eventually the posterior portion of the calcaneum was entirely gone. Sinuses then formed in other parts of the ankle and lower part of the leg. During these thirteen years her health has been precarious, severe illnesses alternating with periods of comparative health. Some weight could be placed upon the toes till about five years ago, since which time the limb has been perfectly helpless and its great weight has made it much of a burden. For that length of time she has been obliged to walk on crutches, and the weight of the limb permitted almost no walking outside the house. She has long been unable to move any of the toes or her ankle in the slightest degree.

When I first saw her, her nutrition was fair, pulse 90 to 100 and temperature normal. The limb from the knee down was enormously enlarged and, at the calf, was thicker than at any part of the thigh. It had the shape precisely of a limb the subject of elephantiasis; the skin, however, was comparatively normal, a little thickened and glazed about the ankle. A number of sinuses opened about the ankle and lower part of the leg, all of which apparently led to the astragalus. The condition of the foot precluded the idea of any conservative operation, the only question being whether to amputate through the leg or at the knee. I amputated about the middle of the leg by antero-posterior flaps, using antiseptic precautions. The muscular tissue at the point of section had entirely disappeared and had been replaced by connective tissue. The flaps cut like salt pork, very heavy, inelastic and were made up entirely of connective tissue, with gaping vessels traversing it, and imbedded in it an occasional tendon. A single sinus in one of the flaps required scraping with the sharp spoon. The bones gave evidence of chronic inflammation. The larger vessels were secured by passing under them a needle armed with catgut, and tying them *en masse.*

Subsequent dissection of the amputated part showed its soft tissues to be in exactly the same condition as existed above; careful search failed to reveal a remnant of muscular fibre in the foot. There was complete disorganization of the ankle-joint, absence of the posterior portion of the calcaneum and beginning disease of the tibia.

The stump healed by primary union and the patient was out of bed on the ninth day. Five months later, her attending physician, Dr. Cyrus McConnell, wrote me that the remaining part of the leg had diminished to about the size of its mate and that her restoration to health had been complete.

As to the pathological condition existing here, I suppose that during these thirteen years of inflammation and caries of the tarsus, there had been a constantly increased supply of blood sent to the foot, and that this had caused the enormous overgrowth of the connective tissue of the limb. Dr. John H. Packard, to whom I related this case, suggested that probably there was also an involvement of the lymph channels as in the so-called elephantiasis. I report this case for the reason that I have no knowledge of any similar one, and the literature at my command does not describe this pathological condition as resulting from carious disease.

Dr. Davis: I think in my experience I have seen a case very similar to Dr. Buchanan's. It was a case of caries of tibia of long standing, in a young woman. I cut down on it and scraped and worked around it in the manner of bone scrapers, without hope of doing her much good. The tissues struck me as being in just the condition that the doctor describes. The part did not heal kindly, and after some weeks the limb was amputated above the knee by my colleague, Dr. Dickson. The description of the tissue makes the two cases very similar.

Dr Allen then made some remarks upon

SYMPATHETIC OPHTHALMIA.

The fact that there is nothing in the whole domain of medicine more important than the saving of the remaining eye to the man who has already lost one, will justify the time I shall consume upon the subject. I will not go into a lengthy discussion of the cause of this affection: some men believe that the germ of suppuration travels from the affected eye through the optic nerve to the sound one: some explain it through the sympathy of the ciliary nerves: whatever the cause, the point I wish to emphasize is, the early removal of the injured ball. "Save the eyeball" is a too frequent cry in cases of injury even after the sight is destroyed. It is a cry that is ominous to the patient. Whether there is a foreign body in the ball or not, (a matter which cannot always be determined), whenever the sight has been destroyed and there is any irritation in the sound eye, early and complete enucleation is the best treatment. The injuries that in my experience are most frequently followed by sympathetic ophthalmia are those through the junction of the cornea and sclerotica, involving the ciliary body. When such an injury exists a sharp watch should be kept for the advent of sympathetic inflammation, and upon its appearance immediate removal of the wounded ball is indicated.

